# Comparative Efficacy of Dapagliflozin and Empagliflozin of a Fixed Dose in Heart Failure: A Network Meta-Analysis

**DOI:** 10.3389/fcvm.2022.869272

**Published:** 2022-04-04

**Authors:** Zepeng Shi, Feng Gao, Wei Liu, Xuezhi He

**Affiliations:** Department of Cardiovascular Surgery, Dalian Municipal Central Hospital, Dalian, China

**Keywords:** heart failure, empagliflozin, dapagliflozin, SGLT-2 inhibitor, prognosis

## Abstract

**Background:**

The efficacy of dapagliflozin and empagliflozin in sodium-glucose cotransport-2 inhibitors (SGLT-2i) in patients with heart failure (HF) has been discovered. However, which drug could improve varied prognostic outcomes has not been elucidated. Hence, we compared their efficacies on the prognostic improvement of HF.

**Methods:**

Databases including PubMed, EMBASE, Scopus, Google Scholars, and the Cochrane Library were searched for all related randomized controlled trials (RCTs) published from inception to 13 October 2021. Network meta-analyses were performed to generate matrices to show the effect size for pairwise comparison regarding all the interventions.

**Results:**

Eventually a total of 11 RCTs were included in this study. For the primary endpoints, dapagliflozin was comparable with empagliflozin in hospitalization for HF, and empagliflozin (OR=0.70, 95%CI: 0.59–0.84) decreased the risk of exacerbation of HF over dapagliflozin. For the secondary endpoints, dapagliflozin was comparable with empagliflozin in cardiovascular (CV) death /hospitalization for HF, and for CV death, dapagliflozin (OR=0.78, 95%CI: 0.65–0.92) significantly reduced mortality over the placebo. For the tertiary endpoints, dapagliflozin (OR=0.80, 95%CI: 0.66–0.98) significantly decreased the mortality over empagliflozin in all-cause death, and neither drug significantly increased the risk of hypoglycemia.

**Recommendations:**

Overall, 10 mg/day dapagliflozin may be the optimal recommendation for its premium and comprehensive effect on improving the prognosis of patients with HF compared to 10 mg/day empagliflozin.

## Introduction

Heart failure (HF), a major public health problem with considerable morbidity and mortality, is often accompanied by various degrees of progressively pathological enlargement of the left ventricular and abnormal cardiac remodeling (1, 2). In recent years, the prevalence of heart failure with preserved ejection fraction (HFpEF) subtype has seized the eyes of scholars in this field. It is generally manifested as left ventricular ejection fraction (LVEF)≥50%, elevated N-terminal pro–B-type natriuretic peptide (NT-proBNP) levels, and definite structural heart disease or diastolic dysfunction in addition to significant signs and symptoms of HF. Its ensuing non-cardiac deaths are more common because of the coexistence of multiple risk factors (age, gender, and BMI) and comorbidities (atrial fibrillation and hypertension) (3). The heart is predominantly remodeled in a centripetal way, so the current triple therapy, angiotensin-converting enzyme inhibitor/angiotensin receptor-neprilysin inhibitor, beta-blockers, and mineralocorticoid receptor antagonist, has limited efficacy in its treatment, and other options are still under exploration (4).

The results of the EMPA-REG OUTCOME trial were the first to demonstrate that empagliflozin in sodium-glucose cotransport-2 inhibitor (SGLT-2i) was not only effective in controlling blood glucose in patients with type 2 diabetes but also in reducing the risk of cardiovascular (CV) death and hospitalization for HF, and the results implied that patients with HF without diabetes may benefit from it as well (5). Undoubtedly, SGLT-2i emerges as a potential therapeutic target for HF. In 2021, the guidelines of the European Society of Cardiology (ESC) and American College of Cardiology (ACC) recommended the addition of dapagliflozin/empagliflozin to the original triple therapy as a new quadruple therapy toward heart failure with reduced ejection fraction (HFrEF), which justifies the two SGLT-2i in treating HFrEF, regardless of diabetes. Moreover, recent studies implied that SGLT-2i might be extended to universal HF more than HFrEF (6).

With its many clinical applications and the publication of large randomized controlled trials (RCTs), SGLT-2i was verified to have CV and renal protective effects in addition to its hypoglycemic mechanism (7). Its diuretic/natriuretic properties may provide additional benefits in terms of reducing circulatory congestion and may reduce the utility of loop diuretics (8). However, there was no head-to-head comparison between dapagliflozin and empagliflozin in terms of improving the prognosis of patients with HF, leaving a paucity of reference in choosing between the two agents. Hence, we conducted a network meta-analysis among dapagliflozin, empagliflozin, and a placebo to find the best agent in benefiting prognosis in patients with universal HF.

## Methods

### Data Sources and Search Strategy

This meta-analysis was conducted and reported in line with Cochrane and PRISMA (Preferred Reporting Items for Systematic review and Meta-Analyses) guidelines (9). Databases including PubMed, EMBASE, Scopus, Google Scholar, and the Cochrane Library were searched for all related randomized controlled trials (RCTs) published from inception to 13 October 2021. The mesh terms are provided in Supplementary Table S1. Manual screening of the reference list of included trials was used to identify any related studies that may have been missed during the search.

### Study Selection and Eligibility Criteria

Inclusion criteria: (1) study subjects were patients with all types of chronic heart failure (CHF, including: HFpEF and HFrEF); (2) the drugs used were dapagliflozin, empagliflozin, and placebo; (3) drug doses of both dapagliflozin and empagliflozin were 10 mg per day; (4) RCTs; and (5) reported at least one prognosis endpoint of interest.

Exclusion criteria: (1) non-RCTs; (2) total number of study cases was <80; (3) drug dosage was inconsistent with the conventional recommended dosage; and (4) full texts were unavailable.

### Definition of CHF

Unlike acute HF, CHF is more stable and the clinical symptoms may not be obvious, but there is also a risk of worsening or decompensation. Integrating the diagnostic criteria of CHF from all included studies collectively, CHF was diagnosed as: (1) patients with New York Heart Association functional class (NYHAFC) II-IV symptoms and LVEF ≤ 50%; (2) NYHAFC I symptoms and LVEF ≤ 40%; (3) LVEF > 40% and 6-min walk test distance ≥ 100 m and ≤ 350 m; (4) NT-proBNP level > 300 pg/ml, or NT-proBNP level > 900 pg/ml in patients with atrial fibrillation.

### Data Extraction and Bias Assessment

A standardized data form was utilized to extract all data by two reviewers independently and Cochrane risk of bias tools was used for RCTs (10). Discrepancy consultation in data extraction and risk of bias assessment was resolved by a third reviewer. We contacted the study sponsor and the investigator, if necessary, to obtain additional trial-level data and to clarify the definition of the results.

### Endpoints of Interest

The primary endpoints were hospitalization for HF and exacerbation of HF [including death/hospitalization for HF, admission to the emergency room, and intravenous diuretics (11)]. The secondary endpoints were CV death/hospitalization for HF and CV death. The tertiary endpoints were all-cause death and hypoglycemia.

### Statistical Analysis

All analyses were performed by the application of STATA version 15.0 and Review Manager software version 5.4. Odds ratio (OR) with 95% confidence intervals (95% CI) was calculated to evaluate the binary variables. If *I*^2^ < 50% and *p* > 0.01, a fixed effect model would be adopted, otherwise a random-effect model would be performed. Network meta-analyses (NMA) were executed according to frequentist framework in Stata software by the random-effects model. Matrices (shown as OR and 95% CI) regarding each endpoint were generated to show the pairwise comparison of all interventions. To enhance the stability of the results, the assessment of both gross and loops inconsistency between direct and indirect comparison were performed. Assessment of small sample effect was performed using funnel plots. *P* < 0.05 was considered to be statistically significant.

## Results

### Eligible Studies and Study Characteristics

**Figure 1:**
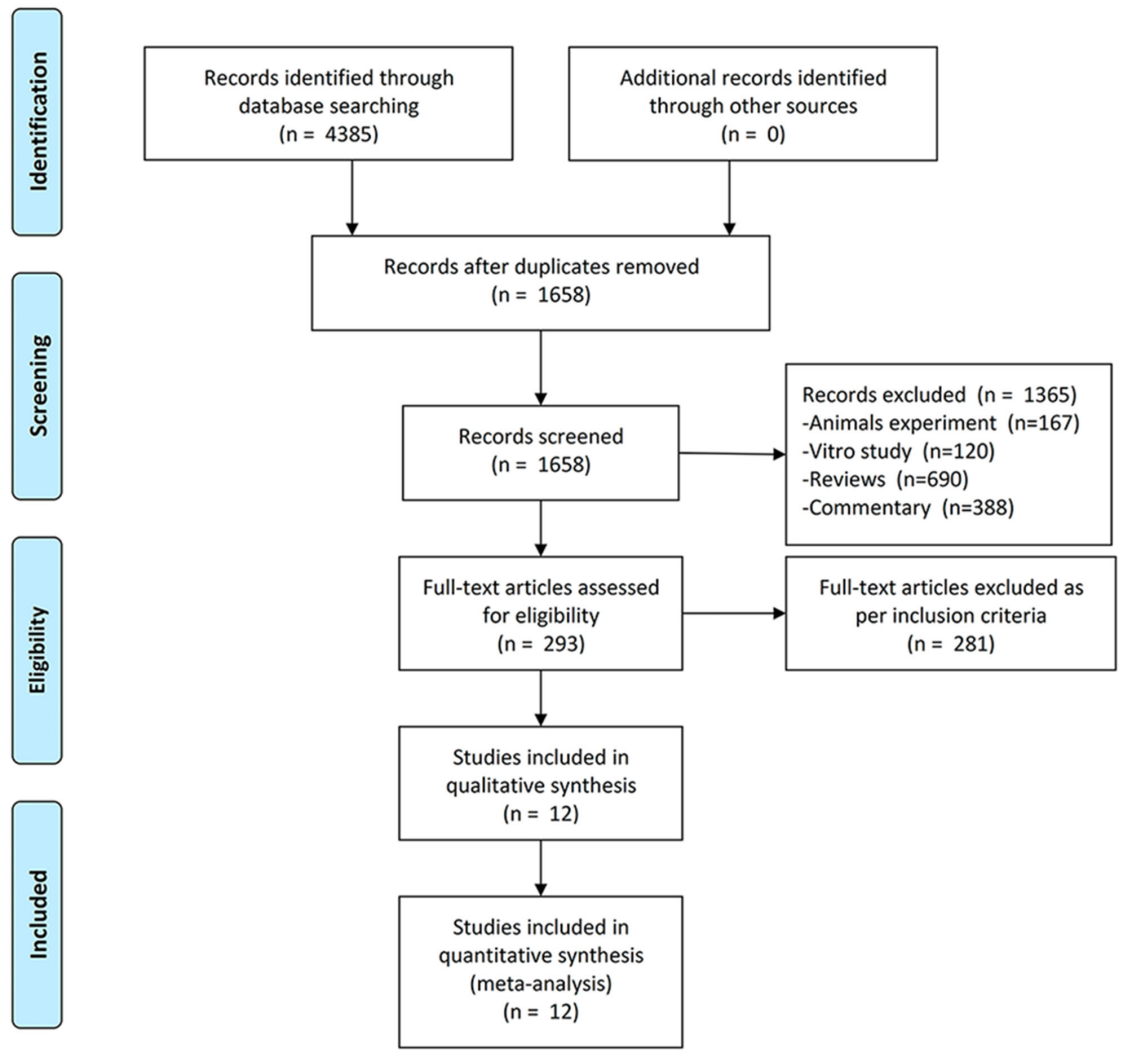
PRISMA flow chart of study selection and identification.

We included the studies from inception to 13 October 2021 and obtained 2,921 publications. After screening and removing duplicates, 293 studies were required to be screened based on inclusion criteria after full-text reading, and finally, 12 studies (2, 6–8, 12–18) containing 11 independent trials were included in this meta-analysis. The screening flow chart is shown in Figure 1. The main characteristics of all included studies have been presented in Table 1 and Supplementary Table S2. Two of the included studies had results from the same clinical trial in a total of two (20) because each study reported results with different tendencies. One study (21) pooled and reported the results of two RCTs.

**Table 1:** Characteristics of population of included studies.

**References**	**Trail name**	**Study groups**	**Age (years)**	**Male (%)**	**NYHAFC (%)**	**LVEF (%)**	**T2D (%)**	**Atrial fibrillation (%)**	**NT–proBNP (pg/ml)**	**Other pharmacological** **therapy (%)**	
Jensen (19)	Empire HF	Empagliflozin (10mg)	64 (57–73)^†^	83.2	II: 75.8; III: 18.9; IV: 0.0	30(25–35)^†^	20.0	37.9	582 (304–1020)^†^	ACEI/ARB: 62.1; ARNI: 32.6	Beta-blocker: 95.8; Loop diuretic: 65.3
		Placebo	63 (55–72)^†^	87.4	II: 81.1; III: 11.6; IV: 0.0	30(25–35)^†^	14.7	35.8	605 (322–1,070)^†^	ACEI/ARB: 68.4; ARNI: 28.4	Beta-blocker: 93.7; Loop diuretic: 62.1
Lee et al. 2020 (15)	SUGAR-DM-HF	Empagliflozin (10 mg)	68.2 (11. 7)^‡^	65.4	II: 71.2; III: 28.8	32.1 (10. 3)^‡^	76.9	NR	1,236 (2342)^‡^	ACEI/ARB: 53.8; ARNI: 40.4	Beta-blocker:88.5; Loop diuretic: 59.6
		Placebo	69.2 (10. 6)^‡^	81.1	II: 83.0; III: 17.0	32.9 (9. 3)^‡^	79.2	NR	1,148 (1905)^‡^	ACEI/ARB: 67.9; ARNI: 28.3	Beta-blocker:94.3; Loop diuretic: 54.7
Abraham et al. 2020 (14)	EMPERIAL-Reduced	Empagliflozin (10 mg)	69 (62.5–77)^†^	77.6	II: 64.7; III: 35.3	30 (24.5–35)^†^	55.8	23.1	2,697.4 (4,357. 5)^‡^	ACEI/ARB: 51.9; ARNI: 39.1	Beta-blocker: 94.9; Loop diuretic: 86.5
		Placebo	70 (62.5–77)^†^	71.2	II: 64.7; III: 35.3	30 (26–36)^†^	64.1	24.4	2,908.5 (4,663. 3)^‡^	ACEI/ARB: 59.0; ARNI: 34.0	Beta-blocker: 94.2; Loop diuretic: 89.1
	EMPERIAL-Preserved	Empagliflozin (10 mg)	74 (62.5–77)^†^	55.4	II: 74.5; III: 24.8	53 (45–58)^†^	54.8	31.8	1,564.3 (2,264. 0)^‡^	ACEI/ARB: 73.2; ARNI: 3.2	Beta-blocker: 89.2; Loop diuretic: 77.1
		Placebo	74 (68–81)^†^	56.3	II: 79.7; III: 20.3	53 (46–59)^†^	47.5	28.5	1,391.3 (1582. 6)^‡^	ACEI/ARB: 75.9; ARNI: 3.8	Beta-blocker: 89.2; Loop diuretic: 66.5
Anker/Packer(HFpEF) et al. 2021 (6, 16)	EMPEROR-Preserved	Empagliflozin (10 mg)	71.8 (9.3)^‡^	55.4	II: 81.1; III: 18.4; IV: 0.3	54.3 (8. 8)^‡^	48.9	51.5	994 (501–1,740)^†^	NR	NR
		Placebo	71.9 (9.6)^‡^	55.3	II: 81.9; III: 17.8; IV: 0.3	54.3 (8. 8)^‡^	49.2	50.6	946 (498–1,725)^†^	NR	NR
Packer et al. /Packer (HFrEF) et al. (2, 8)	EMPEROR-Reduced	Empagliflozin (10 mg)	67.2 (10.8)^‡^	76.5	II: 75.1; III: 24.4; IV: 0.5	27.7 (6.0)^‡^	49.6	35.6	1,887 (1,077–3429)^†^	ACEI/ARB: 70.7; ARNI: 18.3	Beta-blocker: 94.7; Loop diuretic: 86.4
		Placebo	66.5 (11.2)^‡^	75.6	II: 75.0; III: 24.4; IV: 0.6	27.2 (6.1)^‡^	49.8	37.8	1,926 (1,153–3525)^†^	ACEI/ARB: 70.7; ARNI: 18.3	Beta-blocker: 94.7; Loop diuretic: 86.4
Kosiborod et al. (17)		Dapagliflozin (10 mg)	63.6 (7.5)^‡^	64.3	II: 40.4; III: 9.4	NR	13.5	17.0	NR	ACEI/ARB: 90.7; Loop diuretic: 48	Beta-blocker: 84.2
		Placebo	64.9 (7.3)^‡^	61.1	II: 49.0; III: 6.0	NR	14.0	18.0	NR	ACEI/ARB: 75.9; Loop diuretic: 47	Beta-blocker: 79.2
Kato et al. (13)	DECLARE-TIMI 58 (HFrEF)	Dapagliflozin (10 mg)	NR	NR	NR	NR	NR	NR	NR	NR	NR
		Placebo	NR	NR	NR	NR	NR	NR	NR	NR	NR
Nassif et al. (12)	DEFINE-HF	Dapagliflozin (10 mg)	62.2 (11.0)^‡^	72.5	II: 69.5; III: 30.5	27.2 (8.0)^‡^	62.8	43.5	1,136 (668–2,465)^†^	ACEI/ARB: 58.0; ARNI: 35.9	Beta-blocker: 99.2; Loop diuretic: 87.0
		Placebo	60.4 (12.0)^‡^	74.2	II: 62.1; III: 37.9	25.7 (8.2)^‡^	64.4	49.0	1,136 (545–2,049)^†^	ACEI/ARB: 60.6; ARNI: 28.8	Beta-blocker: 93.9; Loop diuretic: 84.1
McMurray et al. (7)	DAPA-HF	Dapagliflozin (10 mg)	66.2 (11.0)^‡^	66.2	II: 67.7; III: 31.5; IV: 0.8	31.2 (6.7)^‡^	41.8	38.6	1,428 (857–2,655)^†^	ACEI/ARB: 84.5; ARNI: 10.5	Beta-blocker: 96.0; Loop diuretic: 93.4
		Placebo	66.5 (10.8)^‡^	77.0	II: 67.4; III: 31.7; IV: 1.0	30.9 (6.9)^‡^	41.8	38.0	1,446 (857–2,641)^†^	ACEI/ARB: 82.8; ARNI: 10.9	Beta-blocker: 96.2; Loop diuretic: 93.5

*NYHAFC, New York Heart Association Function Class; LVEF, left ventricular ejection fraction; GFR, glomerular filtration rate; ACEI, angiotensin-converting-enzyme inhibitors; ARB, angiotensin II receptor blockers; ARNI, angiotensin; NR, no report*.

† *Median (IQR), IQR: interquartile range*.

‡ *Mean (SD), SD: standard deviation*.

### Risk of Bias and Evidence Network

**Figure 2:**
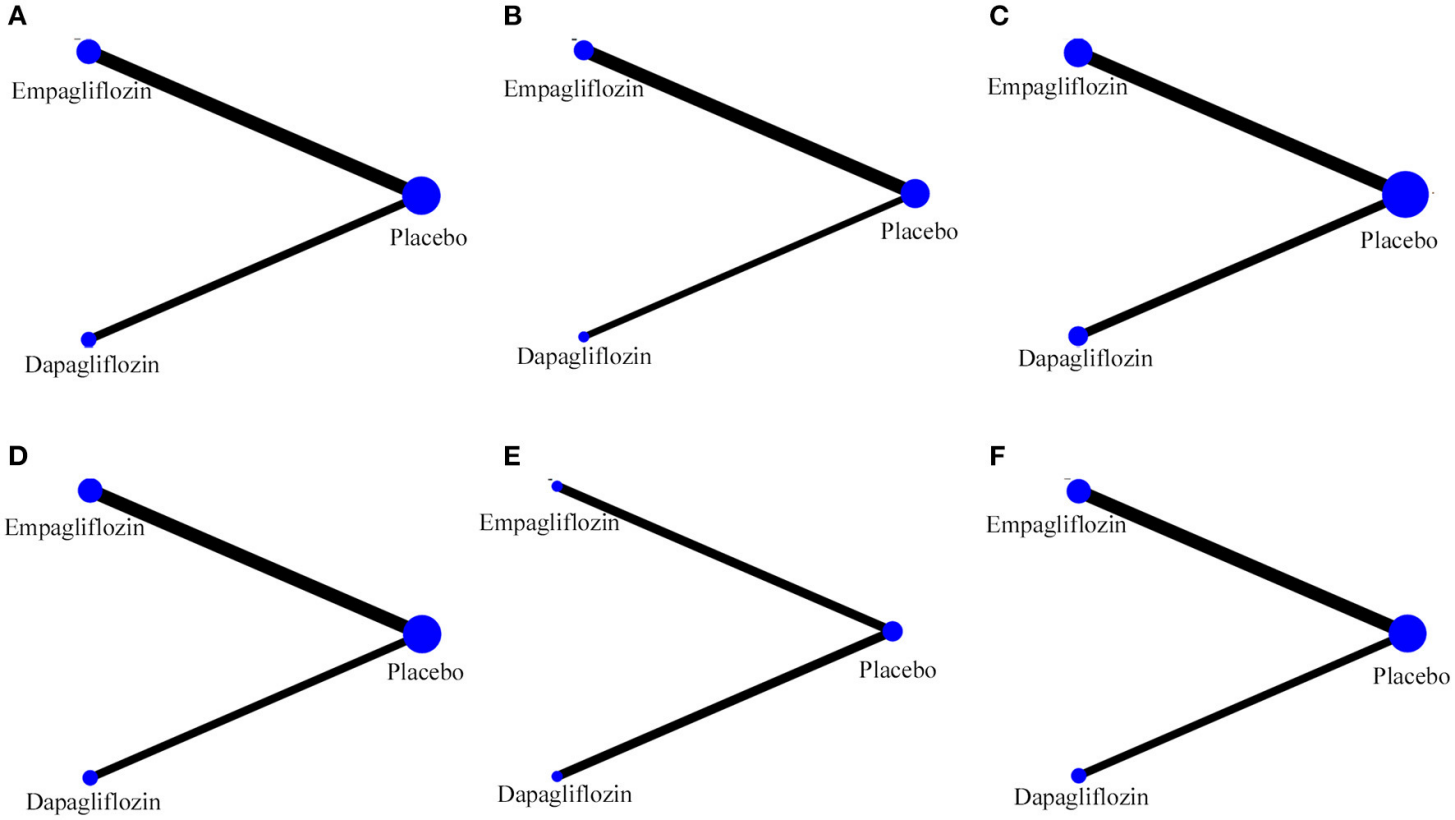
Evidence of network. **(A)** Hospitalization for HF; **(B)** exacerbation of HF; **(C)** all-cause death; **(D)** CV death; **(E)** CV death or hospitalization for HF; **(F)** hypoglycemia.

**Figure 3:**
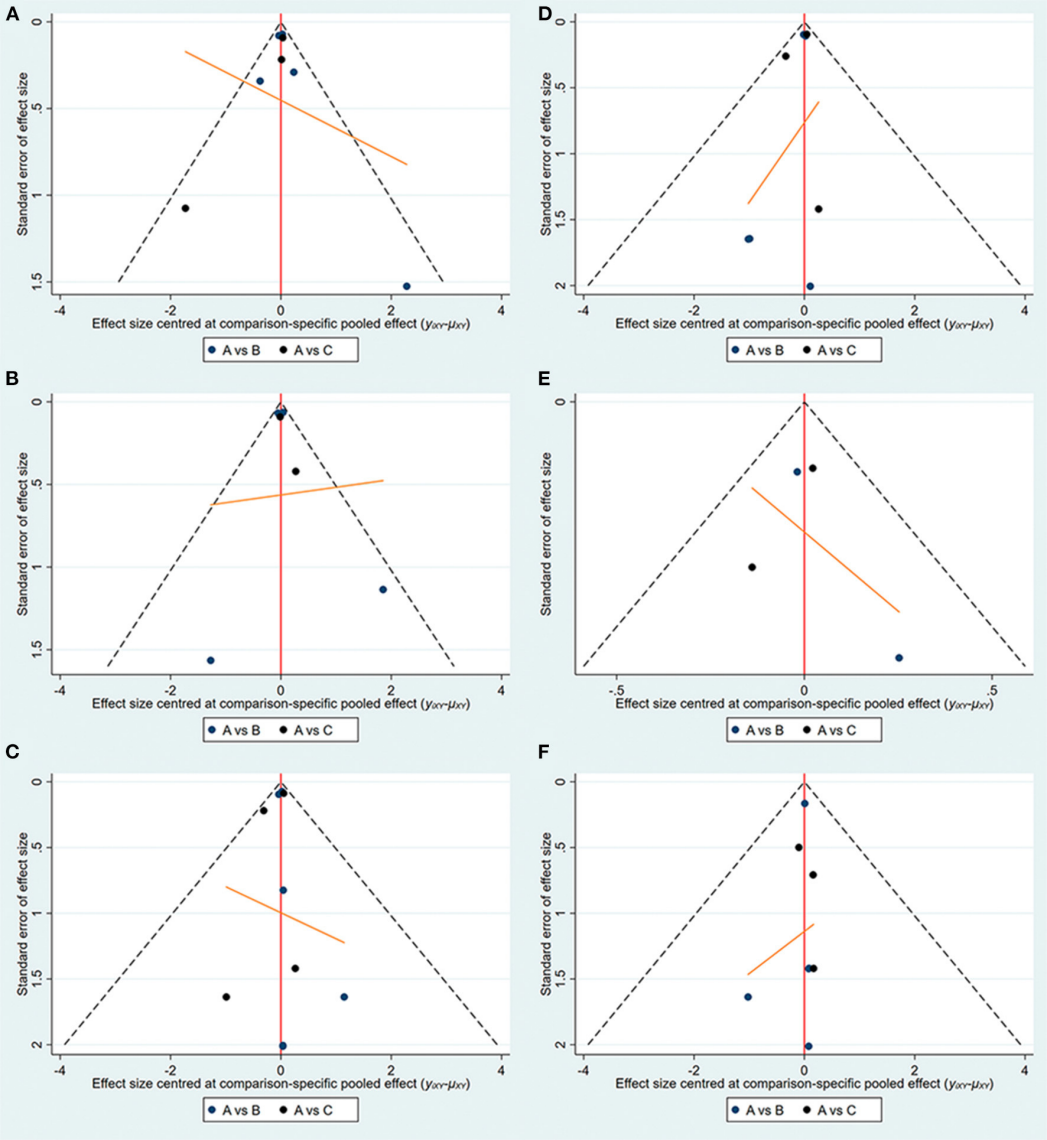
Funnel plots (A:empagliflozin; B:dapagliflozin; C:placebo). **(A)** Hospitalization for HF; **(B)** exacerbation of HF; **(C)** all-cause death; **(D)** CV death; **(E)** CV death or hospitalization for HF; **(F)** hypoglycemia.

The specific details of risk-of-bias assessments of the included studies are shown in Supplementary Figure S1. The evidence of a network is shown in Figure 2, with nodes representing different drug interventions and lines representing direct face-to-face comparisons. The width of the lines represents the number of trials. The size of nodes is related to the sample size of the intervention. The combined funnel plots are shown in Figure 3.

### Direct Meta-Analysis and Network Meta-Analysis

All direct meta-analyses were the comparison of SGLT-2i with placebo (shown in Supplementary Figure S2), and the full matrix results are presented in detail in Table 2.

**Table 2:** Network meta-analysis matrix of results.

**Endpoint**	**Intervention**			
All-cause death		Placebo	Empagliflozin	Dapagliflozin
	Placebo		0.96 (0.86,1.08)	0.77 (0.66,0.91)
	Empagliflozin			0.80 (0.66,0.98)
	Dapagliflozin			
CV death or hospitalization for HF		Placebo	Empagliflozin	Dapagliflozin
	Placebo		0.74 (0.64,0.87)	0.71 (0.62,0.82)
	Empagliflozin			0.95 (0.78,1.17)
	Dapagliflozin			
CV death		Placebo	Empagliflozin	Dapagliflozin
	Placebo		0.90 (0.78,1.03)	0.78 (0.65,0.92)
	Empagliflozin			0.87 (0.69,1.08)
	Dapagliflozin			
Exacerbation of HF		Placebo	Empagliflozin	Dapagliflozin
	Placebo		0.68 (0.62,0.74)	0.70 (0.59,0.84)
	Empagliflozin			
	Dapagliflozin		0.70 (0.59,0.84)	
Hospitalization for HF		Placebo	Empagliflozin	Dapagliflozin
	Placebo		0.76 (0.69,0.84)	0.68 (0.58,0.80)
	Empagliflozin			0.90 (0.75,1.10)
	Dapagliflozin			
Hypoglycemia		Placebo	Empagliflozin	Dapagliflozin
	Placebo		0.92 (0.67,1.27)	0.85 (0.40,1.83)
	Empagliflozin			0.92 (0.40,2.12)
	Dapagliflozin			

*Comparison of treatments:Odd ratios (95% confidence intervals); Effect of intervention in each row compared with intervention in each column*.

### Primary Endpoints

For hospitalization for HF, the OR of empagliflozin *vs*. placebo was 0.76 (95%CI, 0.69–0.84) and dapagliflozin was 0.68 (95%CI. 0.58–0.80). Network meta-analysis of hospitalization for HF showed that the OR of dapagliflozin *vs*.empagliflozin was 0.90 (95%CI, 0.75–1.10). For exacerbation of HF, the OR of empagliflozin *vs*.placebo was 0.68 (95%CI, 0.62–0.74) and dapagliflozin was (0.70; 95%CI, 0.59–0.84). The OR of network meta-analysis of empagliflozin *vs*.dapagliflozin was 0.70 (95%CI, 0.59–0.84).

### Secondary Endpoints

For CV death/hospitalization for HF, the OR of dapagliflozin *vs*. placebo was 0.71 (95%CI 0.62–0.82) and empagliflozin was 0.74 (95%CI, 0.64–0.87). Network meta-analysis of CV death/hospitalization for HF showed that the OR of dapagliflozin *vs*.empagliflozin was 0.95 (95%CI, 0.78–1.17). For CV death, the OR of dapagliflozin *vs*. placebo was 0.78 (95%CI, 0.65–0.92), whereas the OR of empagliflozin was 0.90 (95%CI, 0.78–1.03). The OR of dapagliflozin *vs*. empagliflozin was 0.87 (95%CI, 0.69–1.08).

### Tertiary Endpoints

For all-cause death, the OR of dapagliflozin *vs*.placebo was 0.77 (95%CI 0.66–0.91), whereas the OR of empagliflozin was 0.96 (95%CI, 0.86–1.08). Network meta-analysis of all-cause death showed that the OR of dapagliflozin *vs*.empagliflozin was 0.80 (95%CI, 0.66–0.98). As for hypoglycemia, the OR of dapagliflozin *vs*.placebo was 0.85 (95%CI, 0.40–1.83) and the OR of empagliflozin was 0.92 (95%CI, 0.67–1.27). The OR of dapagliflozin *vs*.empagliflozin was 0.92 (95%CI, 0.40–2.12).

### Consistency and Inconsistency

The overall and loops inconsistency did not exist in all endpoints.

## Discussion

To our knowledge, this was the first network meta-analysis comparing the efficacy of dapagliflozin, empagliflozin, and placebo in patients with universal HF. Unlike previous conventional meta-analysis of SGLT-2i with placebo (22–25), we implemented network comparison to materialized pairwise comparison of dapagliflozin *vs*. empagliflozin with regard to the above endpoints. The dose of the drug was also chosen exclusively as the recommended 10 mg per day. We excluded some studies because they used mixed drug doses and reported confounding results (26). In clinical practice, as the determination of the type of HF was entirely dependent on echocardiographic findings, there were difficulties in classifying patients according to the type of HF when they were in the progress of deteriorating cardiac function. This has a direct impact on the subsequent treatment of the patient and the long-term prognosis. Thus, there is still an urgent need for a new drug that is target specific and has a definite benefit on the prognosis for patients with all types of HF. As mentioned above, recent studies indicated that SGLT-2i might be extended to universal HF more than HFrEF. Thus, this research, as a pilot study to extend them to the full spectrum of HF could be considered as a supplement to the existing guidelines and it is of great clinical value for guiding the medication of all types of HF.

In this work, we compared all the primary endpoints we are concerned about comprehensively. We found that both dapagliflozin and empagliflozin at 10 mg significantly improved the probability of patients being hospitalized for HF compared to the placebo alone and their efficacy was comparable. This might be due to the osmotic diuretic effect of SGLT-2i. It did not inhibit Na-K-2Cl-cotransporter (NKCC2) on macular densa cells and therefore did not activate the RAAS system (27). It also reduced the risk of HF by decreasing urinary magnesium excretion and increasing blood magnesium levels (28).

For exacerbation of HF, we also found that both dapagliflozin and empagliflozin were effective in preventing this type of prognosis in patients relative to the application of placebo. In the meantime, empagliflozin at a dose of 10 mg performed much better. Exacerbation of HF was included in the study as a composite outcome variable, for which part of the mechanism has already been mentioned above. The other part was that SGLT-2i increased hepatic acetone body synthesis, reduced urinary acetone body excretion, increased myocardial acetone body feedstock available, produced more ATP, and reduced myocardial oxygen consumption, which led to a more efficient myocardial energy supply (29). As a result, the activity tolerance of patients with HF can be increased to varying degrees, and the limitations, because of this, in daily living can be effectively reduced.

Abnormalities in blood–lipid metabolism played an important role in predisposing to CV death. Studies have shown that SGLT-2i significantly reduced total cholesterol (TC) and triglyceride (TG) levels and decreased small dense LDL-C, which significantly increased the risk of CV death because of its long intracirculatory cycle, its ability to cross the arterial wall, and its susceptibility to oxidation (30). It had also been established that insulin resistance, circulating levels of leptin, and oxidative stress associated with endothelial cell function were strongly associated with CV death, and SGLT-2i inhibited all of these conditions (31–35). In this meta-analysis, we also found a significant protective effect of dapagliflozin against placebo for CV death, but not for empagliflozin. This was not entirely consistent with the results of previous studies, although it only included two studies (22). We believed that this was because the mean age of empagliflozin was higher than the placebo in the included studies, and that the age factor somewhat reduced its protective effect on CV. Previous studies had identified age as a major risk factor for HF and CV disease, while HF had become one of the leading causes of death in the elderly (36). There was no doubt that the cumulative effect of harmful stimuli (e.g., hypertension and ischemic injury) made the impairment of myocardial reserve more severe in elderly patients due to the limited capacity of the heart to repair and regenerate. Even if this was not the key, the thickening of the ventricular wall, the dilatation of the left atrium, and the increase in the volume of myocardial fibrosis with age all contributed to the objective presence of cardiac dysfunction. Hence, there was a general increase in susceptibility to HF during aging, which in turn drove CV death to some extent. However, at this point in time, after a more objective and comprehensive comparison, the preventive effect of 10 mg of dapagliflozin on CV death is certain. The difference between them suggested that the protective effect of empagliflozin on CV might not be as robust as thought. For the compound variable CV death/hospitalization for HF, we found a significant protective effect of both dapagliflozin and empagliflozin compared to placebo. Otherwise, at the same 10 mg dose, there was no significant difference in the effect of dapagliflozin compared with empagliflozin.

We considered that all-cause death influenced by SGLT-2i was because CV death was so heavily dominant within it, as there was no definitive mechanism to explain its exact effect beyond CV. After analysis, it was found that dapagliflozin at 10 mg was more protective than empagliflozin and placebo. Dapagliflozin showed a stronger protective effect compared to empagliflozin because empagliflozin studies contained a large number of HFpEF patients who suffered from multiple comorbidities (obese, hypertension, atrial fibrillation, etc.) and were often faced with a high risk of non-cardiac deaths (3).

The SGLT-2i lowered blood glucose primarily by lowering the renal glucose threshold and promoting urinary glucose excretion. In patients with HF with normal blood glucose levels, this effect was significantly reduced. In this study, dapagliflozin and empagliflozin at 10 mg orally daily did not increase the risk of severe hypoglycemia compared to placebo, and the effects of both SGLT-2i were generally similar. This was in line with previous research findings (37).

## Limitations

Firstly, to reduce within-group variability in the empagliflozin treatment group, we examined only the difference between the 10 mg dose of empagliflozin and placebo. We observed some 25 mg doses mixed in the treatment groups of some studies, but we had to discard the results of this part of the study because we could not break down its effect in some of the studies. This also made this study miss the exploration of the dose-effect. Secondly, the duration of follow-up was not united in some of the included studies, which somewhat increased the occurrence of time-related reporting bias. Thirdly, the proportions of cardiac function classes were not exactly similar within each study endpoint, making the within-group heterogeneity of some of the results high, but the combined effect did not change reversibly when this type of study was excluded. Finally, due to the limited inclusion of results for comparison, the volume of literature reported for some of the analyses is not sufficient, and further expansion of the literature is needed to verify the validity of the results in this study.

## Observation

In our study, the priority of dapagliflozin in decreasing all-cause death and CV death over empagliflozin and placebo was obvious. Moreover, dapagliflozin showed equivalent effect with empagliflozin on hospitalization for HF and CV death/hospitalization for HF, and both of them were significantly lower than the placebo, despite the fact that empagliflozin showed a clear superior efficacy over dapagliflozin in exacerbation of HF, and none of them increased the risk of hypoglycemia.

## Recommendations

Considering the above results collectively, 10 mg/day dapagliflozin may be the optimal recommendation for its premium and comprehensive effect on improving the prognosis of patients with HF compared to 10 mg/day empagliflozin. However, considering that Empagliflozin study cohorts contained large numbers of HFpEF patients with multiple comorbidities which placed them at high risk for non-CV deaths, further studies are still needed to verify the reliability of the recommendation.

## Data Availability Statement

The original contributions presented in the study are included in the article/Supplementary Material, further inquiries can be directed to the corresponding author.

## Author Contributions

ZS and WL were responsible for screening articles, extracting all data, and applied the Cochrane Collaboration's tool to assess the risk of bias. XH was responsible for theoretical guidance and decision-making in case of disagreement. ZS, FG, and WL were responsible for article writing and data analysis. All authors contributed to the article and approved the submitted version.

## Conflict of Interest

The authors declare that the research was conducted in the absence of any commercial or financial relationships that could be construed as a potential conflict of interest.

## Publisher's Note

All claims expressed in this article are solely those of the authors and do not necessarily represent those of their affiliated organizations, or those of the publisher, the editors and the reviewers. Any product that may be evaluated in this article, or claim that may be made by its manufacturer, is not guaranteed or endorsed by the publisher.

## Abbreviations

SGLT-2i: sodium-glucose cotransport-2 inhibitors
HF: heart failure
RCTs: randomized controlled trials
CV: cardiovascular
OR: odds ratio
NYHAFC: New York Heart Association functional class
LVEF: left ventricular ejection fraction
NMA: network meta-analysis
HFpEF: heart failure with preserveded ejection fraction
HFrEF: heart failure with reduced ejection fraction.
