# Supplementary material for: Comparative Efficacy of Dapagliflozin and Empagliflozin of a Fixed Dose in Heart Failure: A Network Meta-Analysis
Source: Front Cardiovasc Med. 2022 Apr 4;9:869272. doi: 10.3389/fcvm.2022.869272 (PMC9013819; doi:10.3389/fcvm.2022.869272)
Supplement: Supplementary file 1 [file Data_Sheet_1.doc]

**Supplementary Material**

This material has been provided by the authors to give readers additional information about this work.

Supplement to Zepeng Shi, Feng Gao, Wei Liu, Xuezhi He: Comparative Efficacy of Dapagliflozin and Empagliflozin of a fixed dose in Heart Failure: A Network Meta-analysis

**Table of contents:**

Section A: Table S1. Search strategy. ………………………………………………………………2

Section B: Figure S1. Risk of bias assessment. …………………………………………………….6

Section C: Table S2. Characteristics of included studies. ……………………….………………….7

Section D: Figure S2. Direct meta-analysis results. ……………………………………………….10

**Table S1. Search strategy.**

|  |  | Search strategy | Number |
| --- | --- | --- | --- |
| PubMed | #1 | Cardiac Failure OR Heart decompensation OR Decompensation, Heart OR Chronic heart OR Acute heart failure OR Acute decompensated heart failure OR Cardiomyopathy OR Diastolic dysfunction OR cardiac insufficiency OR Diastolic failure OR Diastolic heart failures OR Heart failures, diastolic OR Diastolic heart failure OR Ejection fraction OR End-stage cardiovascular disease OR End-stage heart disease OR Systolic failure OR Heart failures, systolic OR Systolic heart failures OR Systolic heart failure OR Systolic dysfunction OR Ventricular failure OR Ventricular dysfunction OR Dysfunction, ventricular OR Dysfunctions, ventricular OR Ventricular dysfunctions OR Myocardial dysfunction OR Ventricular OR mechanical assist device OR Heart failure OR Heart Decompensation OR Decompensation, Heart OR Heart Failure, Right-Sided OR Heart Failure, Right Sided OR Right-Sided Heart Failure OR Right Sided Heart Failure OR Myocardial Failure OR Congestive Heart Failure OR Heart Failure, Congestive OR Heart Failure, Left-Sided OR Heart Failure, Left Sided OR Left-Sided Heart Failure OR Left Sided Heart Failure OR CardiOvascualr death OR HFpEF OR HF PEF OR HFrEF OR HF REF | 541,865 |
|  | #2 | Empagliflozin OR Inhibitor, SGLT2 OR BI 10773 OR BI10773 OR BI-10773 OR Jardiance OR Dapagliflozin OR Farxiga OR Forxiga OR BMS 512148 OR BMS512148 OR BMS-512148 OR Sodium-Glucose Transporter 2 Inhibitors OR Sodium Glucose Transporter 2 Inhibitors OR Sodium-Glucose Transporter 2 Inhibitor OR Sodium Glucose Transporter 2 Inhibitor OR SGLT-2 Inhibitors OR SGLT 2 Inhibitors OR Gliflozins OR SGLT2 Inhibitors OR Gliflozin OR SGLT-2 Inhibitor OR Inhibitor, SGLT-2 OR SGLT 2 Inhibitor OR SGLT2 Inhibitor OR Inhibitor, SGLT2 | 6927 |
|  | #3 | Clinical trial, phase III OR Phase 3 clinical trial OR Phase III clinical trial OR Phase 3 trial OR Phase III trial OR Phase 3 clinical study OR Phase III clinical study OR Phase 3 study OR Phase III study OR Phase 3 randomized trial OR Phase III randomized trial OR Clinical trial, phase II OR Phase 2 clinical trial OR Phase II clinical trial OR Phase 2 trial OR Phase II trial OR Phase 2 clinical study OR Phase II clinical study OR Phase 2 randomized trial OR Phase II randomized trial OR Phase 2 study OR Phase II study OR Phase 2/3 trial OR Phase II/III trial OR Phase 2/3 clinical study OR Phase II/III clinical study OR Phase 2/3 study OR Phase II/III study OR Phase 2/3 randomized trial OR Phase II/III randomized trial OR Randomized controlled trial OR RCT OR Randomized OR random OR Placebo OR controlled clinical trial OR Random Allocation OR Double-Blind Method OR single-blind method OR single mask OR Double mask | 1,017,442 |
|  | #1 And #2 And #3 | 2021.10.13 | 752 |

**Table S1. (Continued.)**

|  |  | Search strategy | Number |
| --- | --- | --- | --- |
| Embase | #1 | Cardiac Failure OR Heart decompensation OR Decompensation, Heart OR Chronic heart OR Acute heart failure OR Acute decompensated heart failure OR Cardiomyopathy OR Diastolic dysfunction OR cardiac insufficiency OR Diastolic failure OR Diastolic heart failures OR Heart failures, diastolic OR Diastolic heart failure OR Ejection fraction OR End-stage cardiovascular disease OR End-stage heart disease OR Systolic failure OR Heart failures, systolic OR Systolic heart failures OR Systolic heart failure OR Systolic dysfunction OR Ventricular failure OR Ventricular dysfunction OR Dysfunction, ventricular OR Dysfunctions, ventricular OR Ventricular dysfunctions OR Myocardial dysfunction OR Ventricular OR mechanical assist device OR Heart failure OR Heart Decompensation OR Decompensation, Heart OR Heart Failure, Right-Sided OR Heart Failure, Right Sided OR Right-Sided Heart Failure OR Right Sided Heart Failure OR Myocardial Failure OR Congestive Heart Failure OR Heart Failure, Congestive OR Heart Failure, Left-Sided OR Heart Failure, Left Sided OR Left-Sided Heart Failure OR Left Sided Heart Failure OR Cardiovascualr death OR HFpEF OR HF PEF OR HFrEF OR HF REF | 770,780 |
|  | #2 | Empagliflozin OR Inhibitor, SGLT2 OR BI 10773 OR BI10773 OR BI-10773 OR Jardiance OR Dapagliflozin OR Farxiga OR Forxiga OR BMS 512148 OR BMS512148 OR BMS-512148 OR Sodium-Glucose Transporter 2 Inhibitors OR Sodium Glucose Transporter 2 Inhibitors OR Sodium-Glucose Transporter 2 Inhibitor OR Sodium Glucose Transporter 2 Inhibitor OR SGLT-2 Inhibitors OR SGLT 2 Inhibitors OR Gliflozins OR SGLT2 Inhibitors OR Gliflozin OR SGLT-2 Inhibitor OR Inhibitor, SGLT-2 OR SGLT 2 Inhibitor OR SGLT2 Inhibitor OR Inhibitor, SGLT2 | 17,715 |
|  | #3 | Clinical trial, phase III OR Phase 3 clinical trial OR Phase III clinical trial OR Phase 3 trial OR Phase III trial OR Phase 3 clinical study OR Phase III clinical study OR Phase 3 study OR Phase III study OR Phase 3 randomized trial OR Phase III randomized trial OR Clinical trial, phase II OR Phase 2 clinical trial OR Phase II clinical trial OR Phase 2 trial OR Phase II trial OR Phase 2 clinical study OR Phase II clinical study OR Phase 2 randomized trial OR Phase II randomized trial OR Phase 2 study OR Phase II study OR Phase 2/3 trial OR Phase II/III trial OR Phase 2/3 clinical study OR Phase II/III clinical study OR Phase 2/3 study OR Phase II/III study OR Phase 2/3 randomized trial OR Phase II/III randomized trial OR Randomized controlled trial OR RCT OR Randomized OR random OR Placebo OR controlled clinical trial OR Random Allocation OR Double-Blind Method OR single-blind method OR single mask OR Double mask | 1,626,328 |
|  | #1 And #2 And #3 | 2021.10.13 | 1095 |

**Table S1. (Continued.)**

|  |  | Search strategy | Number |
| --- | --- | --- | --- |
| Scopus | #1 | Cardiac Failure OR Heart decompensation OR Decompensation, Heart OR Chronic heart OR Acute heart failure OR Acute decompensated heart failure OR Cardiomyopathy OR Diastolic dysfunction OR cardiac insufficiency OR Diastolic failure OR Diastolic heart failures OR Heart failure, diastolic OR Diastolic heart failure OR Ejection fraction OR End-stage cardiovascular disease OR End-stage heart disease OR Systolic failure OR Heart failure, systolic OR Systolic heart failures OR Systolic heart failure OR Systolic dysfunction OR Ventricular failure OR Ventricular dysfunction OR Dysfunction, ventricular OR Dysfunctions, ventricular OR Ventricular dysfunctions OR Myocardial dysfunction OR Ventricular OR mechanical assist device OR Heart failure OR Heart Decompensation OR Decompensation, Heart OR Heart Failure, Right-Sided OR Heart Failure, Right Sided OR Right-Sided Heart Failure OR Right Sided Heart Failure OR Myocardial Failure OR Congestive Heart Failure OR Heart Failure, Congestive OR Heart Failure, Left-Sided OR Heart Failure, Left Sided OR Left-Sided Heart Failure OR Left Sided Heart Failure OR Cardiovascualr death OR HFpEF OR HF PEF OR HFrEF OR HF REF |  |
|  | #2 | Empagliflozin OR Inhibitor, SGLT2 OR BI 10773 OR BI10773 OR BI-10773 OR Jardiance OR Dapagliflozin OR Farxiga OR Forxiga OR BMS 512148 OR BMS512148 OR BMS-512148 OR Sodium-Glucose Transporter 2 Inhibitors OR Sodium Glucose Transporter 2 Inhibitors OR Sodium-Glucose Transporter 2 Inhibitor OR Sodium Glucose Transporter 2 Inhibitor OR SGLT-2 Inhibitors OR SGLT 2 Inhibitors OR Gliflozins OR SGLT2 Inhibitors OR Gliflozin OR SGLT-2 Inhibitor OR Inhibitor, SGLT-2 OR SGLT 2 Inhibitor OR SGLT2 Inhibitor OR Inhibitor, SGLT2 |  |
|  | #3 | Clinical trial, phase III OR Phase 3 clinical trial OR Phase III clinical trial OR Phase 3 trial OR Phase III trial OR Phase 3 clinical study OR Phase III clinical study OR Phase 3 study OR Phase III study OR Phase 3 randomized trial OR Phase III randomized trial OR Clinical trial, phase II OR Phase 2 clinical trial OR Phase II clinical trial OR Phase 2 trial OR Phase II trial OR Phase 2 clinical study OR Phase II clinical study OR Phase 2 randomized trial OR Phase II randomized trial OR Phase 2 study OR Phase II study OR Phase 2/3 trial OR Phase II/III trial OR Phase 2/3 clinical study OR Phase II/III clinical study OR Phase 2/3 study OR Phase II/III study OR Phase 2/3 randomized trial OR Phase II/III randomized trial OR Randomized controlled trial OR RCT OR Randomized OR random OR Placebo OR controlled clinical trial OR Random Allocation OR Double-Blind Method OR single-blind method OR single mask OR Double mask |  |
|  | #1 And #2 And #3 | 2021.10.13 | 572 |

**Table S1. (Continued.)**

|  |  | Search strategy | Number |
| --- | --- | --- | --- |
| Google scholars | #1 | Cardiac Failure OR Heart decompensation OR Decompensation, Heart OR Chronic heart OR Acute heart failure OR Acute decompensated heart failure OR Cardiomyopathy OR Diastolic dysfunction OR cardiac insufficiency OR Diastolic failure OR Diastolic heart failures OR Heart failure, diastolic OR Diastolic heart failure OR Ejection fraction OR End-stage cardiovascular disease OR End-stage heart disease OR Systolic failure OR Heart failure, systolic OR Systolic heart failures OR Systolic heart failure OR Systolic dysfunction OR Ventricular failure OR Ventricular dysfunction OR Dysfunction, ventricular OR Dysfunctions, ventricular OR Ventricular dysfunctions OR Myocardial dysfunction OR Ventricular OR mechanical assist device OR Heart failure OR Heart Decompensation OR Decompensation, Heart OR Heart Failure, Right-Sided OR Heart Failure, Right Sided OR Right-Sided Heart Failure OR Right Sided Heart Failure OR Myocardial Failure OR Congestive Heart Failure OR Heart Failure, Congestive OR Heart Failure, Left-Sided OR Heart Failure, Left Sided OR Left-Sided Heart Failure OR Left Sided Heart Failure OR Cardiovascualr death OR HFpEF OR HF PEF OR HFrEF OR HF REF |  |
|  | #2 | Empagliflozin OR Inhibitor, SGLT2 OR BI 10773 OR BI10773 OR BI-10773 OR Jardiance OR Dapagliflozin OR Farxiga OR Forxiga OR BMS 512148 OR BMS512148 OR BMS-512148 OR Sodium-Glucose Transporter 2 Inhibitors OR Sodium Glucose Transporter 2 Inhibitors OR Sodium-Glucose Transporter 2 Inhibitor OR Sodium Glucose Transporter 2 Inhibitor OR SGLT-2 Inhibitors OR SGLT 2 Inhibitors OR Gliflozins OR SGLT2 Inhibitors OR Gliflozin OR SGLT-2 Inhibitor OR Inhibitor, SGLT-2 OR SGLT 2 Inhibitor OR SGLT2 Inhibitor OR Inhibitor, SGLT2 |  |
|  | #3 | Clinical trial, phase III OR Phase 3 clinical trial OR Phase III clinical trial OR Phase 3 trial OR Phase III trial OR Phase 3 clinical study OR Phase III clinical study OR Phase 3 study OR Phase III study OR Phase 3 randomized trial OR Phase III randomized trial OR Clinical trial, phase II OR Phase 2 clinical trial OR Phase II clinical trial OR Phase 2 trial OR Phase II trial OR Phase 2 clinical study OR Phase II clinical study OR Phase 2 randomized trial OR Phase II randomized trial OR Phase 2 study OR Phase II study OR Phase 2/3 trial OR Phase II/III trial OR Phase 2/3 clinical study OR Phase II/III clinical study OR Phase 2/3 study OR Phase II/III study OR Phase 2/3 randomized trial OR Phase II/III randomized trial OR Randomized controlled trial OR RCT OR Randomized OR random OR Placebo OR controlled clinical trial OR Random Allocation OR Double-Blind Method OR single-blind method OR single mask OR Double mask |  |
|  | #1 And #2 And #3 | 2021.10.13 | 882 |


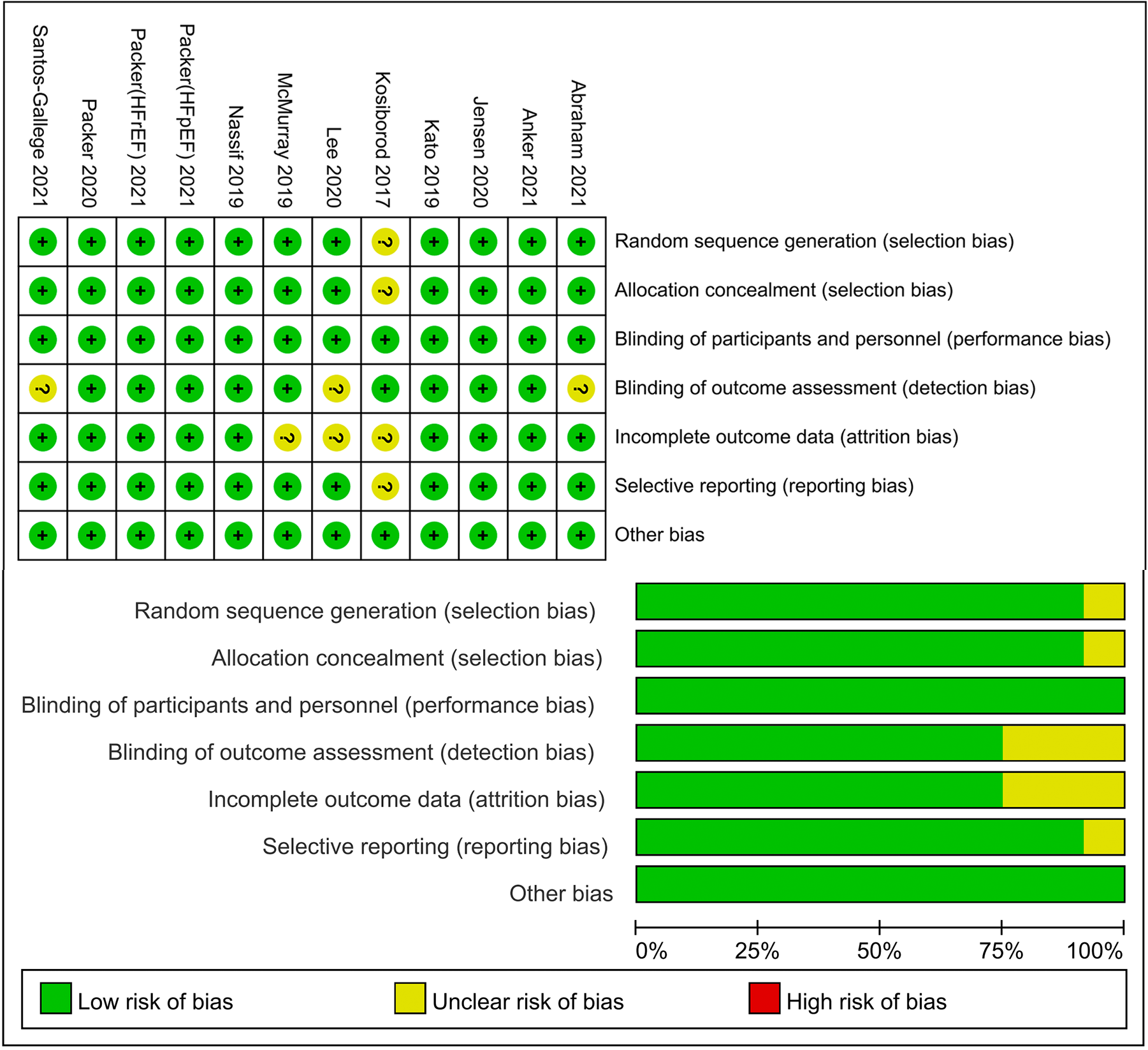


**Figure S1.** Risk of bias assessment.

**Table S2** Characteristics of included studies.

| Author | Year | Trail name | Study design | Main inclusion criteria | Sample size (I/P) | Intervention  (Dose) | Follow-up period | Primary outcome | Conclusion |
| --- | --- | --- | --- | --- | --- | --- | --- | --- | --- |
| Jensen  (19) | 2020 | Empire HF^*^ | Paralell-  group  RCT | NYHA I-III symptoms and HFrEF | 95/95 | Empagliflozin  (10mg once daily) | 3 months | NT-proBNP | Empagliflozin did not change  NT-proBNP in low-risk patients with HFrEF. Daily activity level or health status did not change. |
| Packer (HFpEF)  (16) | 2021 | EMPEROR-Preserved^†^ | Paralell-  group  RCT | NYHA II–IV; HFpEF; NT-proBNP> 300 pg/ml or patients with atrial fibrillation NT-proBNP>  900 pg/ml. | 2997/2991 | Empagliflozin  (10mg once daily) | 20 months | Deaths, hospitalizations for any reason, outpatient events | In patients with HFpEF, empagliflozin produced a reduction in the risk and severity of worsening heart failure events. |
| Packer  (2) | 2020 | EMPEROR-Reduced^*^ | Paralell-  group  RCT | Chronic heart failure (NYHA II- IV) with HFrEF | 1863/1867 | Empagliflozin  (10mg once daily) | 18 months | A composite of cardiovascular deaths or hospitalizations for worsening heart failure | Empagliflozin group had a lower risk of CV death or hospitalization for HF. |
| Packer  (HFrEF)  (8) | 2021 | EMPEROR-Reduced^*^ | Paralell-  group  RCT | Chronic heart failure (NYHA II- IV) with HFrEF | 1863/1867 | Empagliflozin  (10mg once daily) | 18 months | The composite of cardiovascular death or hospitalization for heart failure | Empagliflozin reduced the risk of inpatient and outpatient worsening heart failure events. |
| Santos-Gallego  (18) | 2021 | EMPATROPISM^§^ | Paralell-  group  RCT | >18 years, NYHA II-III,  LVEF<50%, stable symptoms and medical therapy>3 months. | 42/42 | Empagliflozin  (10mg once daily) | 6 months | LVEDV, LVESV | Empagliflozin improved LV volumes, LV mass, LV systolic function, functional capacity, and quality of life. |
| Lee  (15) | 2020 | SUGAR-DM-HF^*^ | Paralell-  group  RCT | >18 years with HFrEF and T2D (history or undiagnosed diabetes with HbA1c≥48 mmol/mol or prediabetes HbA1c 39-47 mmol/mol. | 52/53 | Empagliflozin  (10mg once daily) | 10 months | LVESVi to BSA, LV GLS | Empagliflozin reduced LV volumes in patients with HFrEF and type 2 diabetes or prediabetes. |

**Table S2** (Continued.)

| Author | Year | Trail name | Study design | Main inclusion criteria | Sample size (I/P) | Intervention  (Dose) | Follow-up period | Primary outcome | Conclusion |
| --- | --- | --- | --- | --- | --- | --- | --- | --- | --- |
| Anker  (6) | 2021 | EMPEROR-Preserved^†^ | Paralell-  group  RCT | NYHA II–IV; HFpEF; NT-proBNP> 300 pg/ml or, for patients with atrial fibrillation NT-proBNP>  900 pg/ml. | 2997/2991 | Empagliflozin  (10mg once daily) | 20 months | A composite of cardiovascular death or hospitalization for heart failure | Empagliflozin reduced the combined risk of cardiovascular death or hospitalization for patients with HFpEF. |
| Abraham  (14) | 2021 | EMPERIAL-Reduced^*^  EMPERIAL-Preserved^†^ | Paralell-  group  RCT | ≥18 years; NYHA II–IV; Structural heart disease; Hospitalization for heart failure within 12 months; 6MWTD≤350 m; NT-proBNP>300 pg/ml for patients without atrial fibrillation, >600 pg/ml for patients with atrial fibrillation; Clinically stable. | 156/156  157/158 | Empagliflozin  (10mg once daily) | 3 months | 6MWTD | Empagliflozin was well tolerated in HF patients. |
| Kosiborod  (17) | 2017 |  | Paralell-  group  RCT | T2D and CVD, hypertension, or moderate renal impairment (eGFR ≥30 to <60 mL/min/1.73 m2  ); NYHA I-III | 171/149 | Dapagliflozin (10mg once daily) | 8 months | A composite endpoint of adjudicated CV death, myocardial infarction, stroke, hospitalization for unstable angina, and adverse events. | Dapagliflozin produced clinically meaningful reductions in HbA_1_c, weight, and SBP in  patients with T2DM and HF, and was well tolerated. |
| Nassif  (12) | 2019 | DEFINE-HF^*^ | Paralell-  group  RCT | HF for at least 16 weeks, LVEF≤40%; NYHA II-III | 131/132 | Dapagliflozin (10mg once daily) | 3 months | Mean NT-proBNP and meaningful improvement in  health status  or NT-proBNP | Patients with HFrEF used dapagliflozin over 12 weeks did not affect mean NT-proBNP but improved patients’ HF-related  health status and natriuretic peptides. |

**Table S2** (Continued.)

| Author | Year | Trail name | Study design | Main inclusion criteria | Sample size (I/P) | Intervention  (Dose) | Follow-up period | Primary outcome | Conclusion |
| --- | --- | --- | --- | --- | --- | --- | --- | --- | --- |
| Kato  (13) | 2019 | DECLARE-TIMI 58 (HFrEF^‡^) | Paralell-  group  RCT | Patients with T2D with either atherosclerotic CV disease or its’ multiple risk factors, and with a creatinine clearance ≥60 mL/min, NYHA I-III. | 318/353 | Dapagliflozin (10mg once daily) | 4.2 years | CV death, hospitalization for HF, and all-cause  mortality. | Dapagliflozin reduced hospitalization for HF in patients with and without HFrEF, and  reduced CV death and all-cause mortality in patients with HFrEF. |
| McMurray  (7) | 2019 | DAPA-HF^*^ | Paralell-  group  RCT | >18 years, NYHA II-IV,  LVEF≤40%. | 2373/2371 | Dapagliflozin (10mg once daily) | 18.2 months | Worsening heart failure or death from cardiovascular causes. | Among patients with HFrEF, the risk of worsening heart failure or death from CV causes was lower among those who  received dapagliflozin than among those who received placebo. |

RCT: randomized controlled trial. HF: heart failure. HFrEF: HF with reduced ejection fraction. HFpEF: HF with preserved ejection fraction. NYHA: New York Heart Association Functional Class. I/P: intervention/placebo. NR: not reported. T1D: type 1 diabetes mellitus. T2D: type 2 diabetes mellitus. eGFR: estimated glomerular filtration rate. GFR: glomerular filtration rate. HbA1c: glycated hemoglobin. LV: left ventricle. LVEF: left ventricular ejection fraction. BMI: body mass index. CV: cardiovascular: CVD: cardiovascular disease. BP: blood pressure. PCCWP: pulmonary capillary wedge pressure. CI: cardiac index. MRI: magnetic resonance imaging. LVESVi: left ventricular end-systolic volume indexed. BSA: body surface area. LV GLS: left ventricular global longitudinal strain. LVEDV: LV end-diastolic volume. LVESV: LV end-systolic volume. 6MWTD: 6-minute walk test distance.

^*^In this study, HFrEF was defined as LVEF < 40%. ^†^ In this study, HFpEF was defined as LVEF≥40%. ^‡^In this study, HFrEF was defined as LVEF < 45%. ^§^In this study, LVEF < 50%

**
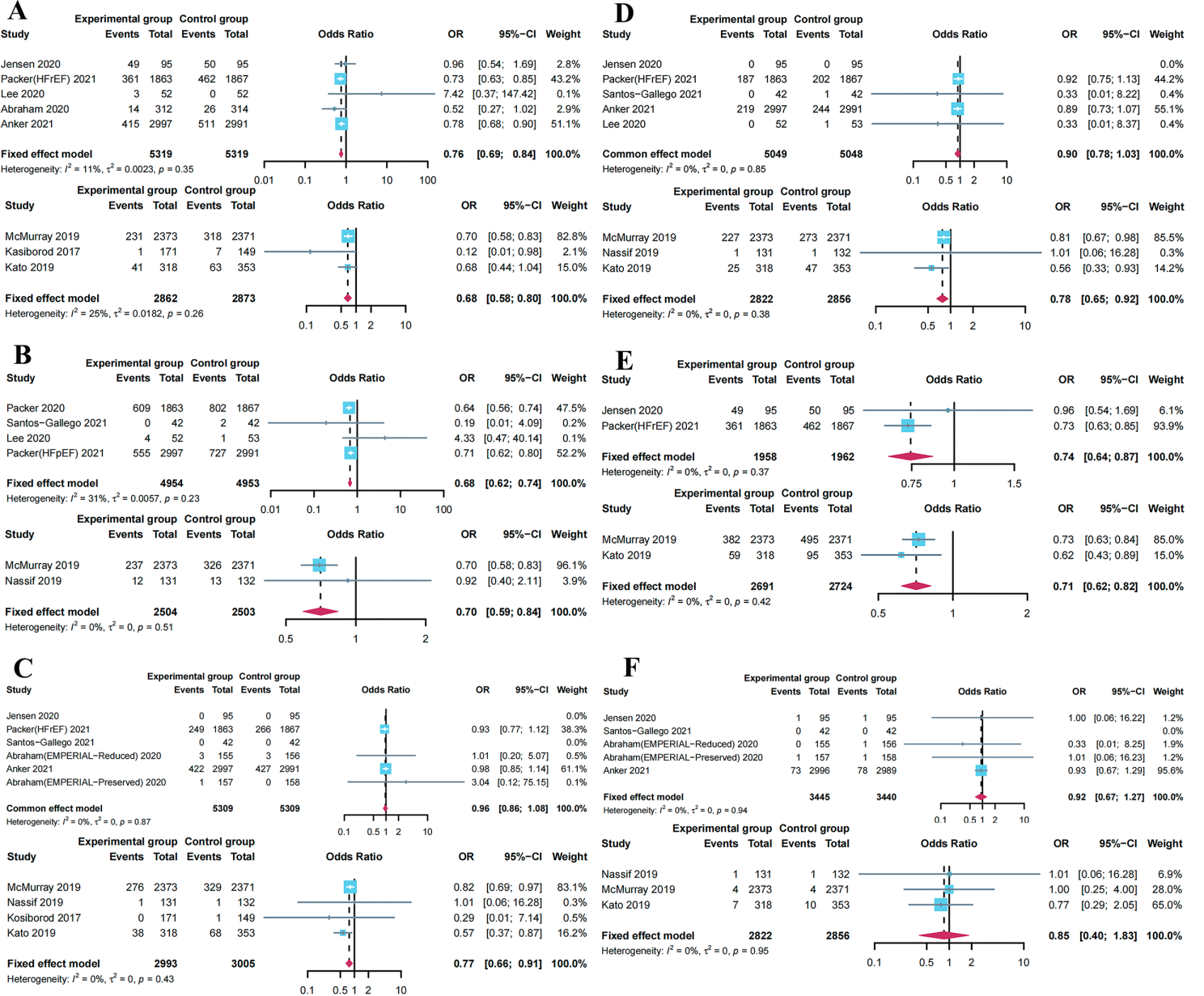
**

**Figure S2.** Direct meta-analysis results (SGLT-2i vs placebo). A) Hospitalization for HF; B) Exacerbation of HF; C) All-cause death; D) CV death; E) CV death or hospitalization for HF; F) Hypoglycemia.
